# Increased Umbilical Cord PAI-1 Levels in Placental Insufficiency Are Associated with Fetal Hypoxia and Angiogenesis

**DOI:** 10.1155/2016/7124186

**Published:** 2016-01-19

**Authors:** Maxim D. Seferovic, Madhulika B. Gupta

**Affiliations:** ^1^Department of Obstetrics and Gynecology, Baylor College of Medicine, Houston, TX 77030, USA; ^2^Children's Health Research Institute, Departments of Biochemistry and Pediatrics, University of Western Ontario, London, ON, Canada N6C 2V5

## Abstract

In intrauterine growth restriction (IUGR), a subset of pregnancies undergoes placental vascular dysregulation resulting in restricted blood flow and fetal hypoxemia. Altered transcription of hypoxic regulated plasminogen activator inhibitor 1 (PAI-1) has been associated with pregnancy complications and angiogenic regulation. Here we assessed circulating PAI-1 as an indicator of placental insufficiency. Venous umbilical PAI-1 of hypoxemic (V*p*O_2_ 20 versus 35 mmHg, *p* < 0.0001) placental insufficient pregnancies (resistance index 0.9 versus 0.63, *p* < 0.05) (*n* = 18) was compared to controls (*n* = 12). PAI-1 was increased (~10-fold, *p* < 0.001) and had a positive predictive ratio of 6.7. Further, PAI-1 levels correlated to blood oxygen (*r* = −0.68, *p* < 0.0001). The plasma's angiogenic potency measured* in vitro* was associated with umbilical cord blood PAI-1 levels (*r* = 0.65, *p* < 0.01). This association was attenuated by PAI-1 inhibiting antibody (*p* < 0.001). The results demonstrate PAI-1 as a potential marker of placental insufficiency and identify its close association with pathological hypoxia and angiogenesis in a subset of growth restricted pregnancies.

## 1. Introduction

The pregnancy complication intrauterine growth restriction (IUGR) occurring in 5–10% of pregnancies is a major contributor to our healthcare burden. Newborns with severe IUGR (<1,500 g at term) have a 70-fold greater mortality rate. Perhaps as critically, these IUGR infants are at much greater risk for long term chronic disease including negative cognitive and behavioral outcomes, as well as increased risk of obesity and cardiovascular and metabolic disease [1–5]. Biomarkers associated with the pathology could help better predict those at risk of developing hypertension, heart disease, and diabetes later in life.

In affluent societies the primary cause of IUGR is a subset of patients that have deficient maternal fetal exchange of gasses and nutrients stemming from a compromised placental vasculature. Specifically, a reduction in vessel volume and degree of branching of the chorionic villous tree increases the vascular resistance to blood flow of the fetal-placental circulation in the later stages of pregnancy [6]. This condition termed placental insufficiency leads to slowed or even arrested fetal umbilical artery diastolic flow compromising maternal/fetal exchange, causing fetal hypoxemia and acidosis. The biochemical regulatory basis of angiogenesis in placental insufficiency has therefore been of considerable research interest in uncovering the pathological mechanisms.

As an inhibitor of urokinase-type and tissue-type plasminogen activators, plasminogen activator inhibitor-1 (PAI-1) has long been implicated in thrombotic disease. PAI-1 is the primary physiological inhibitor of plasminogen activation in blood, which either promotes or prevents vascular remodeling processes; PAI-1 has been determined to be a proangiogenic factor [7]. There is significant correlation of circulating PAI-1 levels to tumor vascularization and metastases [8–10]. Elevated plasma PAI-1 is a prognostic biomarker for poor outcome in breast cancer [11–15]. It functions through extracellular matrix (ECM) regulation, which drives cell migration and angiogenesis [16].

In pregnancy PAI-1 is also secreted by the syncytiotrophoblast, where its increased mRNA levels have been histologically determined in preeclamptic placentas with IUGR [17]. Its elevated maternal levels have previously been associated with clinical metrics of fetal blood supply [18]. PAI-1 levels have been variably reported as decreased [19] or unchanged [20] in IUGR pregnancies. Few studies have separated etiological differences of IUGR pregnancies, controlled for conditions like preeclampsia, which though it has a large overlap in diagnosis is likely an independent pathology [17, 20, 21], and have PAI-1 regulatory alterations of their own [22, 23]. Further, PAI-1 is downstream of numerous regulatory pathways, including vascular endothelial growth factor A (VEGF) and fibroblast growth factor 2 (FGF-2) that are both implicated in the normal development of the placental vasculature [24, 25]. These cytokines in turn are themselves altered in IUGR [26–29], presumably influencing PAI-1 expression. Finally, the finding that, like VEGF, PAI-1 is downstream of a hypoxic response element and therefore is regulated via hypoxic inducible factor 1 (HIF-1) mediated induction suggests a potential association between its expression and the placental insufficiency pathology [30].

Levels of plasma PAI-1 in placental insufficiency are therefore downstream of competing molecular regulatory mechanisms related to hypoxia. Hypoxia is the main stimulus for angiogenesis by signaling through hypoxia-inducible transcription factors. Since fetal hypoxia is variable from normal to life threatening in placental insufficiency, we hypothesized that PAI-1 levels at birth could act as a marker of placental insufficiency and further that the altered levels would associate with the hypoxic and angiogenic changes that underlay the pathology. From a restrictive subset of very severe IUGR pregnancies that have a demonstrated compromised vasculature and placental insufficiency as measured by umbilical Doppler, we set out to test if PAI-1 is in fact changing in the fetal-placental plasma in placental insufficiency, and also whether those changes are sufficient to influence angiogenesis.

## 2. Materials and Methods

### 2.1. Subject Recruitment and Plasma Collection

Pregnant women were recruited with informed consent from St. Joseph's Hospital, London, Canada, with approval by the University of Western Ontario Research Ethics Board. Women with severe IUGR with placental insufficiency as diagnosed by umbilical artery Doppler were included in the study (*n* = 18). Mothers with placental insufficiency were otherwise healthy and absent for other significant pregnancy complications so as to minimize the biological variability associated with diverse etiological factors underlying IUGR. Specific exclusion criteria included multiple gestations, preeclampsia, placental abruption, fetal congenital abnormalities, fetal or placental infection, and maternal diabetes, as well as drug and alcohol use. Randomly selected gestational age matched pregnancies were chosen that also excluded all major obstetrical complications listed other than preterm birth. Because IUGR babies are at high risk for fetal morbidity or mortality, these babies are frequently delivered preterm. In order to gestational age-match the IUGR group, we selected either preterm normal birth weight for gestational age or term normal birth weight for gestational age babies, on a balanced basis. These control pregnancies were otherwise free of obstetrical complications (*n* = 12).

Women with suspected IUGR had estimated fetal weights determined by fetal biometric ultrasound measurements, with estimated fetal weight well below the tenth percentile for GA to ensure selection of small for gestational age infants that was confirmed to be below the 10th percentile birth weight following delivery. Umbilical Doppler was measured by a maximum of three days prior to delivery to determine the placental resistance in IUGR pregnancies and diagnose placental insufficiency. Since no IUGR was suspected or diagnosed in most healthy term pregnancies, Doppler measurements were not indicated within the last week of pregnancy and therefore were not performed nor abstracted for this study. All IUGR patients were confirmed to be placental insufficient by umbilical Doppler (Table 1). Gestational age was determined by last menstrual date of mothers or the first trimester ultrasound crown rump length. The groups were therefore separated by those with a low birth weight given their gestational age, or percentile birth weight (IUGR defined as <10th percentile), as the additional subdiagnosis of placental insufficiency (Table 1).

Immediately following delivery the umbilical cord was clamped and fetal blood extracted from the fetal umbilical cord vein by venipuncture in EDTA coated tubes. One tube of blood was analyzed at St. Joseph's Hospital as part of normal neonatal care, where blood gas values were immediately determined by the clinical laboratory in hospital using a Radiometer ABL 500 Blood Gas Analyzer. A second tube from the same sampling was centrifuged at 3500 ×g for 15 min at 4°C, and the plasma supernatants were saved for subsequent analysis at −80°C. Venous and arterial oxygen levels were then determined from the same venipuncture as the plasma samples collected immediately following delivery. Oxygen values were abstracted from fetal charts.

The birth weight percentiles were calculated following delivery using standardized growth charts [31]. A subset of the IUGR pregnancies diagnosed with placental insufficiency and confirmed growth restricted based on birthweight were included. Though below the 10th percentile is indicative of IUGR, of the 18 IUGR pregnancies included, all but 2 were confirmed to be <3rd percentile, and all but 4 were below the 1st percentile. The low percentile likely reflects a combination of selection of clear cases of IUGR and isolation of the severe subtype of placental insufficiency as confirmed by umbilical Doppler. The restrictive subset of low percentile IUGR pregnancies with demonstrated compromised vasculature ensured isolation of a clear etiological subset of IUGR babies.

All control pregnancies included were >25th percentile for gestational age and gender. Pathological examination of the placentas was undertaken at St. Joseph's Hospital. Based on review of the pathological reports and other information in the patient charts, some subjects were retroactively excluded for diverse medical complications including fetal congenital anomalies, major infarcts, or maternal antenatal hypertension or suspected infection. The umbilical blood gas levels used in this paper were also abstracted from the chart for 16 of 18 IUGR and 12 of 12 controls. The blood samples for protein analysis and for blood gas analysis were taken at the same time and from the same venipuncture into separate tubes. Due to the rarity of the cases based on restrictive inclusion and exclusion criteria, both vaginal and Caesarean section deliveries were included, and the effects of delivery mode as well as gestational age were assessed.

### 2.2. HUVEC Culture and Angiogenic Assays

Human umbilical vein endothelial cells (HUVECs) (Lonza, Basel, Switzerland) were grown in Endothelial Growth Media (Lonza) supplemented with 10% FBS, using CELL+ growth surface coated flasks (Sarstedt, Numbrecht, Germany). For treatments, HUVECs were plated on ECMatrix (Millipore, MA, USA) at a density of 1 × 10^4^ or 3 × 10^4^ cells per well (96-well plate) with 50 *μ*L of FBS-free media. HUVECs have played a major role as a model system for the study of the regulation of the endothelium in the development of atherosclerotic plaques and angiogenesis. In this study, the plasma collected was added to Endothelial Base Media (Lonza, Basel, Switzerland) (FBS-free) for a final concentration of 1% in 100 *μ*L of media to determine its effects of tubule formation using HUVECs. The cells were allowed to grow within ~4–6 hours as per the* in vitro* Angiogenesis Assay Kit protocol (Millipore, MA, USA). For PAI-1 neutralizing antibody inhibitor treatments, mouse anti-PAI-1 antibodies (BD Transduction Laboratories, Franklin, NJ, USA) were added to the plasma in 30 *μ*L of base media and incubated at RT for 1 hour. Cells were then added to the tubes, and incubated for a further 10 minutes prior to plating. Three bright field images were taken using an inverted microscope. Leica FireCam software was used to capture the images (v3.4, Leica Microsystems, Wetzlar, Germany). The tube length was quantified using Axiovision software (v4.7.1, Carl Zeiss, Germany), and branches were counted manually as per the Angiogenesis Assay Kit and as described previously [32].

### 2.3. Protein Measurements and Statistical Evaluations

All total protein quantifications were by Bradford method (BioRad). FGF-2, VEGF, and PAI-1 were measured by an immunological-based fluorescent multiplex assays (Human CDV1, Millipore, Billerica, MA, USA) using a Bio-Plex 200 system (BioRad), which utilizes Luminex xMAPTM fluorescent bead-based technology (Luminex Corp., Austin, TX). Levels were automatically calculated from standard curves using Bio-Plex Manager software (v4.1.1, BioRad). All statistics were done using Prism 6 (Graph Pad Software Inc., CA, USA).

To compare means, *t*-tests for parametric or Mann-Whitney test for nonparametric data as appropriate was used following an *F* test. One-way ANOVAs were used to measure significance across multiple means with Tukey's* post hoc* test to compare between means. Changing proteins' levels were compared to blood oxygen levels, as well as other quantitative criteria abstracted from the clinical charts, and the degree of angiogenesis measured in the angiogenic assay. Multicorrelational analysis was via Pearson's correlation matrices. To correct for multiple comparisons, a *Q* of 0.05 was calculated at *p* < 0.01 which was considered the false discovery threshold of correlational significance.

The receiver operating characteristic curve (ROC) was determined by comparing the groups separated by the gold standard of umbilical Doppler diagnosis of >95th percentile of umbilical artery resistance index and confirmed growth restriction as measured by percentile birth weight. The true positive and false positive prediction rate were plotted and compared against umbilical oxygen levels of the blood which upregulate PAI-1 and are indicators of fetal hypoxemia and morbidity but are unreliable as they are transient and rapidly changing as a marker of placental insufficiency. The area under the ROC was used to assess the accuracy of prediction, where above 0.8 was considered good.

## 3. Results

Samples were collected from small for gestational age babies that were first identified by routine ultrasound, lack of weight gain, and other screening metrics, as likely IUGR possibly caused by placental insufficiency. A subset of pregnancies subsequently confirmed to be placental insufficient by umbilical artery Doppler were included. Because a large proportion of these IUGR babies are delivered preterm to minimize the risk of morbidity or mortality, they were gestational age matched preterm or term controls as the appropriate GA matched control. Whether preterm or term, controls were appropriate growth for their gestational age (~50th percentile birth weight) compared to small for gestational age (~3%) (Table 1). We therefore separated those with a low birth weight given their gestational age as a result of placental insufficiency, from those that had birth weight that was appropriate for their gestational age (controls), regardless of preterm or term birth (birth weight percentile, Table 1).

At birth, effluent placental blood samples were collected from the umbilical cord vein. Mothers selected for the study, spanned in gestational age from 26.5 to 39 weeks. Healthy or often preterm controls were selected as gestational age matched controls that were otherwise free of complications. IUGR babies were confirmed to be growth restricted with birth weight percentiles <10% for gestational age, and placentae weighing ~125 g less on average (*p* < 0.05) (Table 1). All pregnancies were diagnosed with placental insufficiency based on umbilical artery Doppler. The resistance was abnormally high for gestational age for all cases of IUGR, demonstrating the vascular pathology of the study group, while data was available for only 5 of 12 controls that were all normal (Table 1). Although the maternal systolic blood pressure was slightly elevated for the placental insufficiency group, the overall normal blood pressure (BP) levels reflect the exclusion of preeclampsia subjects.

**Figure 1:**
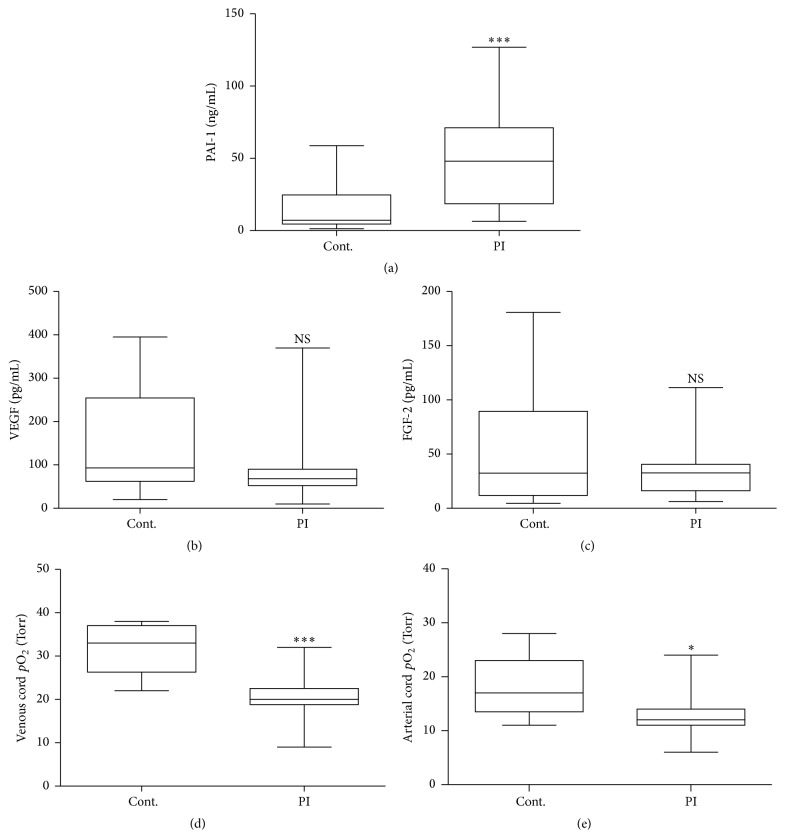
Fetal umbilical cord plasma levels of PAI-1 are elevated in PI. Fetal umbilical cord plasma levels of PAI-1 and regulators of its transcription were compared between PI. (a) PAI-1 is greatly increased in the fetal circulation in placental insufficiency (Mann-Whitney test, ^*∗∗∗*^ *p* < 0.001). Total VEGF (b) and FGF-2 (c) levels were unchanged, while oxygen levels in both the venous (d) and arterial (e) umbilical cord were decreased in placental insufficiency (*t*-test, ^*∗*^ *p* < 0.05, ^*∗∗∗*^ *p* < 0.001). Blood gas measurements were performed in hospital by the clinical laboratory for 16/18 PI and 11/12 control subjects as part of routine postnatal care. Multiplex immunofluorometric assays were used to measure protein changes from the same samples. Box plots indicate median, interquartile range, and whiskers total range.

The effluent placental blood collected from the umbilical cord was analyzed for PAI-1 levels, and also for its cytokine regulators VEGF and FGF-2, using a fluorescent multiplex immunoassay. PAI-1 was dramatically elevated (~10-fold, *p* < 0.001) in placental insufficiency compared to control group (Figure 1(a)). No corresponding change was detected for total VEGF and FGF-2 (Figures 1(b) and 1(c)). The levels of umbilical oxygen (abstracted from the fetal clinical chart), however, were significantly reduced in placental insufficiency (both venous and arterial, *p* < 0.001 and *p* < 0.05, resp.) (Figures 1(d) and 1(e)), further indicating the restricted exchange as a result of the placental vascular pathology.

Although venous cord blood oxygen levels were significantly different between control and placental insufficiency, there was no difference attributable to delivery mode, in either control or placental insufficiency groups, of either venous or arterial oxygen levels (Supplemental Figure  1; see Supplementary Material available online at http://dx.doi.org/10.1155/2016/7124186). Likewise the delivery mode had no effect on the elevated levels of PAI-1, VEGF, and FGF-2. PAI-1 and VEGF levels also did not change with gestational age; however FGF-2 decreased with gestational age in controls only (Supplemental Figure  2), which is consistent with expectations [33]. Similarly, as PAI-1 is upregulated by corticosteroids* in vitro* we examined the effect of subjects who received steroid treatments as prophylactic (<34 weeks) compared with those who did not (>34 weeks). It was found that the PAI-1 was increased in the <34-week group 42 ng/mL versus 30 ng/mL; however this was not significant (*p* = 0.36). This indicates that although there may be some effect, it may be lost in the larger changes associated with placental insufficiency.

**Figure 2:**
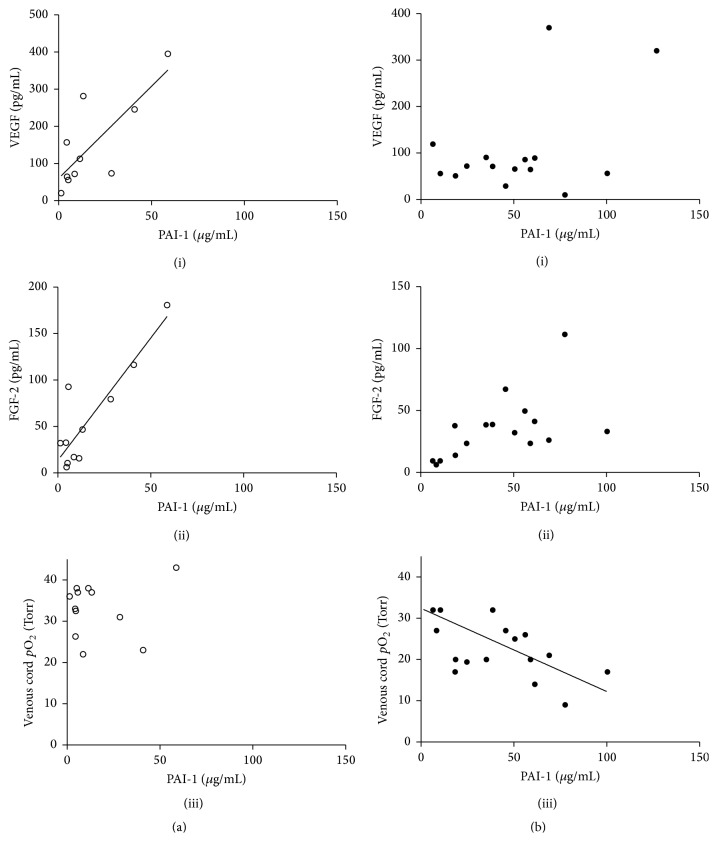
PAI-1 correlates to the expression of PAI-1 regulators in the fetal umbilical cord blood plasma. (a) In control pregnancies, PAI-1 levels are dependent on total VEGF (i) and FGF-2 (ii) levels (*r* = 0.78, *p* < 0.01, and *r* = 0.89, *p* < 0.001, resp.), but not venous umbilical oxygen (iii). (b) Conversely in PI pregnancies, PAI-1 expression has no relationship to total VEGF (i) or FGF-2 (ii) levels, but is instead dependant on ambient oxygen levels (iii) (*r* = −0.68, *p* < 0.0001) (open: control, closed: PI).

**Figure 3:**
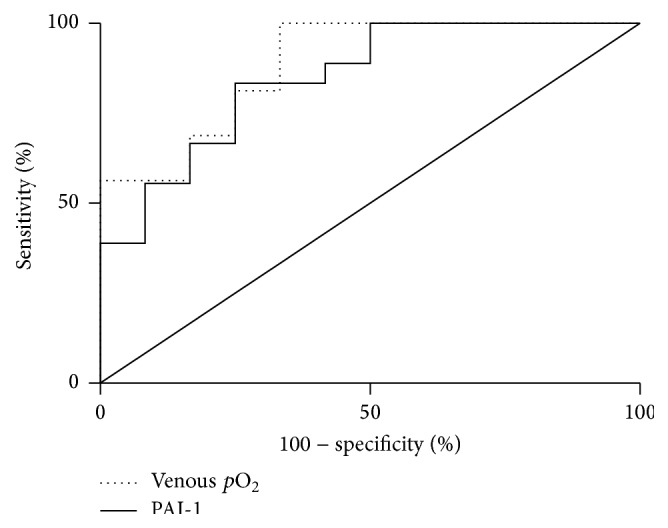
Receiver operator characteristic curves comparing PAI-1 to oxygen as markers of placental insufficiency.

Levels of VEGF or FGF-2, though unchanged between placental insufficiency and control, correlated to the elevated concentrations of PAI-1 in control pregnancies (Figure 2(a) (i) and (ii)). The relationships were strongly dependent, where *r* = 0.78 (*p* < 0.01) and *r* = 0.89 (*p* < 0.001) for VEGF and FGF-2, respectively. However, this dependency did not carry over to the placental insufficiency group (Figure 2(b) (i) and (ii)). Instead, for placental insufficiency pregnancies, the higher concentration of PAI-1 in plasma was dependent on the oxygen levels (*r* = −0.68, *p* < 0.0001), where there was no relationship in controls (Figure 2(a) (iii) versus 2(b) (iii)). ROC reveal PAI-1 to be approximately as sensitive and as specific an indicator of placental insufficiency as venous blood oxygen level following delivery (Figure 3). The area under the curve was 0.847 for PAI-1 compared to 0.885 for venous cord blood oxygen. A cut-off value was determined at >43.3 ng/mL, where PAI-1 has a sensitivity of 55.6% and a specificity of 91.7% corresponding to a true positive likelihood ratio of 6.7.

**Figure 4:**
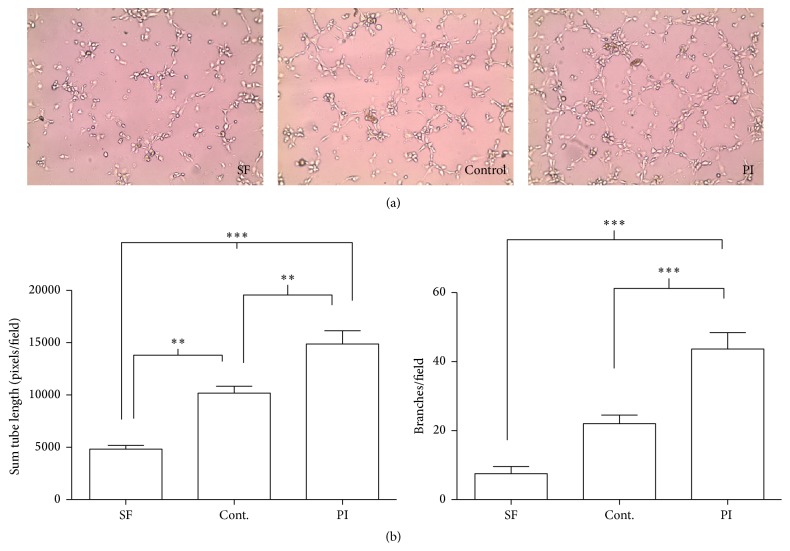
Umbilical artery plasma from PI pregnancies has increased angiogenic potency. Angiogenic tube formation assay of HUVECs plated onto extracellular matrix protein gel in the presence of 1% venous umbilical cord plasma from PI and control. Assays were individually performed on PI (*n* = 10) and control (*n* = 10) samples in duplicate experiments. Each sample was gestational age matched within 5 days. (a) Representative fields are shown from serum-free control (SF), control pregnancy plasma, and PI plasma. (b) Quantification was using the standard method of calculating total tube length of all cord formation, and the total number of branches per field. Results were determined by averaging across four fields per well, with triplicate wells per experiment (one-way ANOVA, ^*∗∗*^ *p* < 0.01, and ^*∗∗∗*^ *p* < 0.001). Standard error of the mean is indicated.

PAI-1 is considered to be a key molecule in thrombotic vascular diseases that is upregulated in hypoxia. Given that PAI-1 is an angiogenic mediator, we measured the effects of the venous cord blood on angiogenesis, using an* in vitro* HUVEC tube formation assay. HUVEC cells were plated on extracellular matrix and were then subjected to 1% venous umbilical cord plasma of placental insufficiency and control pregnancies in a basal media. Representative images from the assays are shown in Figure 4(a). The amount of angiogenic potency of the placental insufficiency plasma relative to control was compared using total length of tube formation and the number of branches per field as metrics using a standard angiogenesis assay kit [34]. The placental insufficiency plasma induced 1.5-fold greater tube length (*p* < 0.01) and a 2-fold greater degree of tube branching (*p* < 0.001) compared to the control group (Figure 4(b)).

**Figure 5:**
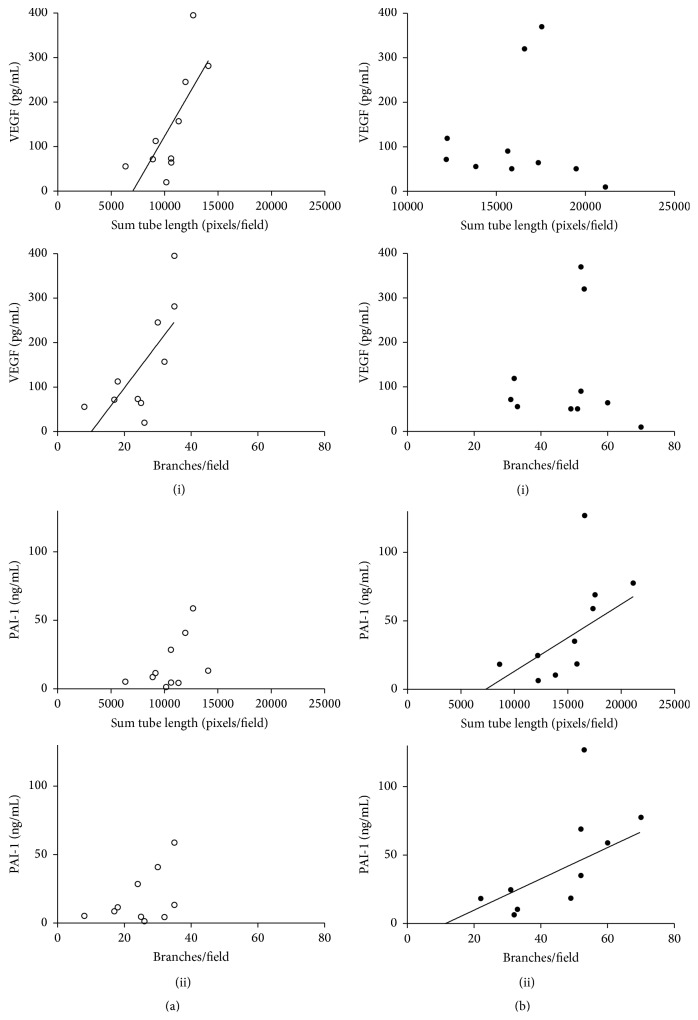
Angiogenic potency correlates to PAI-1 levels in PI. The angiogenic potency of the plasma that was measured (Figure 4) was correlated to PAI-1 and VEGF. (a) In control pregnancies, the angiogenic potency was strongly dependent on total VEGF levels (i) in both measurements of tube formation and branching (*r* = 0.71 and 0.74, *p* < 0.05, resp.). However there was no relationship to PAI-1 (ii). (b) Conversely in pregnancies with PI, measure of angiogenesis was unrelated to VEGF levels (i) and, instead, correlated to circulating PAI-1 levels (ii) for both tube formation and branching (*r* = 0.65 and 0.67, *p* < 0.01 and <0.05, resp.) (open: control, closed: PI).

When the degree of angiogenic induction by the plasma was correlated against the proteins' concentration, it was revealed unsurprisingly that it correlated significantly to total VEGF (but not FGF-2) levels in the plasma in both measurements of tube formation and branching (*r* = 0.71, and *r* = 0.74, *p* < 0.05, resp.) (Figure 5(a) (i)). However, the correlation again did not carry over to the placental insufficiency group (Figure 5(b) (i)). Instead, for placental insufficiency pregnancies, the angiogenic potency correlated to PAI-1 levels for both tube formation and branching (*r* = 0.65, *p* < 0.01, and *r* = 0.67, *p* < 0.05, resp.), where there was no relationship in controls (Figure 5(a) (ii) versus 5(b) (ii)).

**Figure 6:**
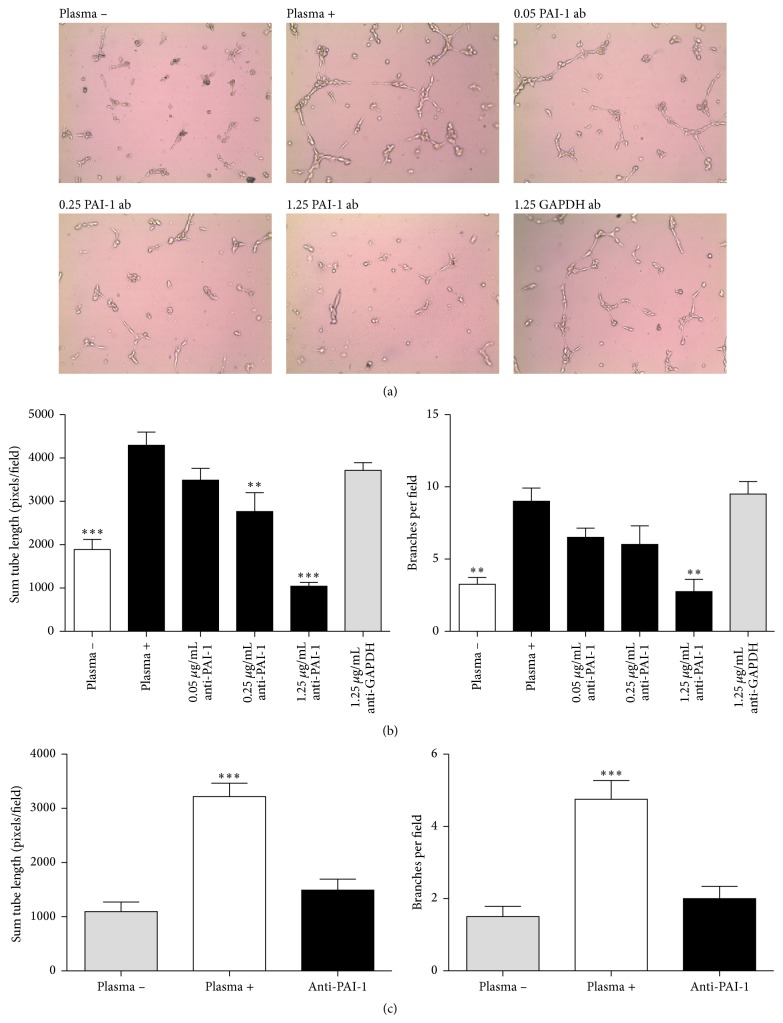
PAI-1 neutralization results in angiogenic suppression. HUVEC angiogenic tube formation assay on extracellular matrix in the presence of 1% venous umbilical cord plasma from PI pregnancies. PAI-1 neutralizing antibody was added in indicated amounts (*μ*g/mL) as well as plasma-free, and anti-GAPDH antibody negative controls and the total tube length and branching quantified following incubations. (a) Representative 100x magnified fields are shown. (b) Significant differences from the plasma + treatment were determined by one-way ANOVA. PAI-1 inhibition attenuated angiogenic simulation measured by both total tube length and branching. (c) Experiments were repeated with six fetal umbilical cord plasma samples from pregnancies with PI, using 1 *μ*g/mL of PAI-1 inhibiting antibody. Standard error of the mean is indicated (one-way ANOVA) (^*∗∗*^ *p* < 0.01 and ^*∗∗∗*^ *p* < 0.001).

To determine if the angiogenic changes observed were directly related to PAI-1 levels, the umbilical cord plasma from placental insufficiency pregnancies was subjected to various concentrations of a PAI-1 activity neutralizing antibody. Representative fields from the tube formation assay are shown in Figure 6(a). Quantifying the inhibition showed the changes to be significant; 0.25 *μ*g/mL of antibody was sufficient to suppress tube formation (*p* < 0.05), while 1.25 *μ*g/mL of anti-PAI-1 inhibited the total tube length and the branches per field ~2-fold (*p* < 0.001 and *p* < 0.01, resp.) (Figure 6(b)), which was similar to HUVECs grown in the absence of plasma. The finding was reproducible across six umbilical plasma samples tested (Figure 6(c)). The inhibition of tube formation with neutralizing antibody implicates umbilical cord plasma PAI-1 in the upregulated angiogenesis of placental insufficiency.

## 4. Discussion

The findings of this study identify PAI-1 as a potential marker of placental insufficiency and associate its levels directly with not only the degree of hypoxia but also angiogenic regulation changes that may underlay the pathology. Its predictive levels were similar to that of routinely assessed hypoxia. Indeed PAI-1 levels were in close step with the degree of hypoxia but furthermore were also strongly associated with angiogenic changes that may underlay the pathology. The fact that PAI-1 levels and angiogenesis in turn are associated with hypoxia in placental insufficiency, and not its molecular angiogenic regulators VEGF and FGF-2, indicates upregulation may be largely through HIF-1 mediated mechanisms. Inhibiting PAI-1 attenuated the plasma's stimulation of tube formation in a dose dependent manner, demonstrating PAI-1's central significance to angiogenesis as a circulating factor. Given the known significant role of PAI-1 in angiogenesis, and its role as a clinical biomarker of tumor vascularization, the extreme upregulation (~10-fold) of PAI-1 in the plasma may have very significant consequences for vascular regulation in the placenta. These data add to evidence of a key role of PAI-1 in the pathophysiology of IUGR. Further the results provide a basis for PAI-1 levels to be assessed for use as a marker of placental disease following delivery for newborns determined to be IUGR.

Blood gasses following delivery are typically used to assess acidosis or alkalosis at delivery which may be acute. In this study, patients were assessed as parentally insufficient and the reduced umbilical oxygen levels and high carbon dioxide observed are in most cases reflective of severely reduced exchange in the placenta. The venous umbilical cord* p*O_2_ as measured approximates that of placental oxygen, correlating 0.80 to the placental levels [35]. PAI-1 expression is known to be upregulated by VEGF and FGF-2 [16]. The levels of both of these cytokines in plasma correlated strongly with PAI-1 levels (Figure 2(a) (i) and (ii)) (which in turn were strongly related to angiogenic potency (Figure 5(a))), however, only for controls. In normal pregnancy the expression of angiogenic cytokines may well be responsible for the largest degree of PAI-1 expression and its corresponding influence on angiogenesis.

It is surprising that VEGF did not increase in hypoxic conditions of placental insufficiency, nor did its levels correlate with the degree of hypoxia in the blood (data not shown). Previous studies have variably reported placental expression of VEGF to increase [27, 36], decrease, or not change in IUGR [37, 38]. It has been suggested that placental insufficiency can result in hyperoxia localized to the placental villi [39, 40] which may limit or decrease VEGF expression from the villous trophoblast layer [41–43]. Whether placental growth factor (PlGF), which is increased in circulation in well oxygenated conditions of pregnancies, upregulates PAI-1 is unknown, though it may plausibly do so through vascular endothelial growth factor receptor 1 (FLT-1) binding [44].

PAI-1, like VEGF, is also directly upregulated via HIF-1 in hypoxia [30], and PAI-1 levels did increase very significantly in correlation with decreasing venous oxygen levels (Figure 2(b) (iii)) in placental insufficiency pregnancies. This association with oxygen and the lack of association with regulatory cytokines in placental insufficiency suggests that the large increase in PAI-1 levels observed may be through direct HIF-1 mediated transcription. Similarly the lack of strong association with corticosteroids suggests that although corticosteroid administration to assist in lung maturation in severely preterm birth pregnancies may have some effect on fetal-placental PAI-1 expression, there was no significant difference observed in our cohort. Furthermore, the changes were not of a magnitude that would suggest they would mask the 10-fold mean change observed in placental insufficiency. Nevertheless, investigations of the expression of PAI-1 should account for cortisone administration as a potential confounder to a marker in severe preterm pregnancies.

PAI-1 proangiogenic action has been suggested to be through modifying the balance of plasmin degradation of the ECM providing a stable ECM platform for cell migration, proliferation, and vessel maturation [45]. It additionally has a vitronectin binding function that may block the binding of endothelial integrins in cell migration [46]. Mice lacking PAI-1 expression stave off invasion and vascularization of transplanted malignant tumors [8]. It is required for postischemic injury angiogenesis in the retina in a murine model of angiogenesis [7]. Recent studies have found that PAI-1 knockout mice have transiently reduced maternal and fetal-placental vasculature [47].

The range of PAI-1 spanned ~1–50 ng/mL in normal pregnancy, typically <10 ng/mL. With hypoxic placental insufficiency it was ~50 ng/mL on average and ranged 3x higher (Figure 1(a)). In line with this, the best cut-off separation determined from the ROC was at 43.3 ng/mL. In context, circulating PAI-1 levels ranging from 50 ng/mL or higher represent a significant departure from normal physiological levels which may influence function. Indeed PAI-1 levels were strongly associated with angiogenesis (Figure 5(b) (ii)), and its inhibition was sufficient to largely reverse plasma-induced angiogenesis* in vitro* (Figure 6). The effects of a near-total inhibition of PAI-1 are not dissimilar to some control pregnancies which were <1 *μ*g/mL. Circulating concentrations of >30 ng/mL have significant physiological effect to nearly triple the risk of breast cancer mortality by comparison [48]. PAI-1 is strongly associated with tumor vascularization, where its increased levels have been associated with poor prognosis [9, 10, 13]. Late gestation fetal-placental vascular conditions in placental insufficiency are analogous to conditions in some breast tumors in that there is vascular dysregulation, inflammation, hypoxia, and altered regulation of VEGF and other genes via HIF-1 [49].

IUGR has multiple etiology, and the stringency in excluding nonidiopathic placental insufficiency and other complications of our patient selection (detailed in Section 2) is a critical strength of this study. Studied samples represent the most severe cases of IUGR, with very low birth weight percentile, and were critically verified to have significantly elevated resistance index for gestational age to only include confirmed vascular malformations that are hallmark of placental insufficiency (Table 1) [50, 51]. Although most controls did not have Doppler assessment of placental insufficiency, the lack of any indication of IUGR as determined by routine ultrasound and regular symphysis fundal height examination and confirmed by birth weight percentile assessment rules out the possibility of an abnormal Doppler finding in controls, as indeed these tests were not medically indicated for these patients. For this reason it was possible to determine ROC confidently against a “gold standard.” This was not only helpful in isolating a type case where the greatest changes might be expected but also reduced the likelihood of inclusion of normal percentile cases due inaccuracies in estimated gestational ages. Controls were selected based on gestational age matching for the overall effect of a very well controlled study group of severe idiopathic IUGR with placental insufficiency. However the molecular effects of an enrichment of spontaneous preterm birth subjects in controls versus indicated preterm birth in placental insufficiency must be considered. A review of the literature offers no conclusive links of changing plasma PAI-1 levels to initiation of parturition, though it is involved in proteolytic events in the decidua.

Conversely, the stringent exclusion criteria may possibly have directed collection efforts towards the most evident placental insufficiency cases that may potentially be more severe than average that could affect the true predictive value of PAI-1. Placental insufficiency is among the most severe of the etiologies, a comparison between large cohorts of mixed etiologies would best determine its predictive value. Nevertheless PAI-1's 10-fold mean separation from control and strong correlations with pathological factors like hypoxia are strong indications of its potential specificity. Finally, our measurements of PAI-1 were necessarily postnatal. Since the genesis of the pathology may initiate largely between 7 and 25 weeks [52], our experiments are likely capturing the effects but not causes of a restricted and reduced vasculature. To what extent the severe changes in PAI-1 initiate the pathology is therefore left unassessed.

For tumor development, it has been demonstrated that elevated circulating PAI-1 has significant implications as a biomarker even within the normal physiological range. Its predictive value has been tied to its function as a central angiogenic mediator. Here we have discovered circulating PAI-1 levels in venous cord blood as highly elevated and strongly associated with the hypoxemia borne of the vascular pathology of placental insufficiency. The levels correlated directly to the plasma's increased angiogenic potency. Although, in metabolic substrate restrictive conditions, circulating protein levels changes may be adaptive [53–55], it is speculated that PAI-1 may be contributing to the vascular pathology in placental insufficiency by promoting vascular resistance. In summary, our findings identify PAI-1's potential utility as a postnatal marker of placental disease. Its clinical use may supplement existing blood gas measurements that are rather more indicative of acute acidosis than placental function* per se*.

## 5. Conclusions

PAI-1 is predictive of placental dysfunction in placental insufficiency. Its levels are strongly related to hypoxia where it may be upregulated directly through HIF-1 mediated mechanisms. Increased plasma PAI-1 levels contribute to upregulated angiogenic mechanisms. A large prospective cohort comparing PAI-1 levels together with the findings of umbilical artery Doppler would determine its utility in retrospectively identifying patients born following a pregnancy with placental dysfunction for etiological classification in IUGR.

## Supplementary Material

We sought to determine if the changes in umbilical blood proteins observed were related either to the onset of labor or to the gestational age of the infant. No significant changes were observed for PAI-1, VEGF, or FGF-2 in association with labor in either group. Similarly, umbilical cord oxygen levels were unaffected in our subjects (Supplemental Figure 1). There was also no change in PAI-1 or VEGF associated with GA. There was a significant negative correlation of FGF-2 with GA for controls that could not be seen in IUGR patients (supplemental Figure 2). FGF-2 has previously been reported to gradually decrease in fetal circulation after 20 weeks in normal pregnancy (Hill et al., 1995).
Hill, D.J., Tevaarwerk, G.J., Arany, E., Kilkenny, D., Gregory, M., Langford, K.S., and Miell, J. (1995). Fibroblast growth factor-2 (FGF-2) is present in maternal and cord serum, and in the mother is associated with a binding protein immunologically related to the FGF receptor-1. *J. Clin. Endocrinol. Metab*. 80 :1822–1831.

## Figures and Tables

**Table 1:** Characteristics of IUGR patients with PI and gestational age matched control pregnancies.

	Control	PI
Subjects (*n*)		
Male	7	13
Female	5	5
Total:	12	18
Birth weight (g)	2172 (723)	1469^*∗*^ (637)
Gestational age (wk)	33.1 (4.1)	34.1 (3.8)
Birth weight percentile	51 (20)	1.5^*∗∗∗*^ (3.0)
Caesarean/vaginal	6/6	12/6
Maternal age (y)	27 (6)	29 (6)
Placental weight (g)	502 (156)	372^*∗*^ (151)
Placental resistance^+^	0.63 (0.09)	0.90^*∗*^ (0.23)
Maternal BP		
Systolic	114 (10)	129^*∗*^ (16)
Diastolic	71 (8)	78 (12)
APGAR (5 min)	8.8 (0.4)	7.9 (2.3)

Comparison of means was by *t*-test or Mann-Whitney test following a normality test (^*∗*^ *p* < 0.05, ^*∗∗∗*^ *p* < 0.001). Standard deviation is indicated in brackets. Fetal blood samples were collected immediately following either vaginal or Caesarean section delivery from the clamped umbilical cord vein by venipuncture in EDTA coated tubes. Blood gas values were measured in the clinical lab and values abstracted from fetal charts.

^+^Placental resistance index from the last umbilical cord Doppler ultrasound prior to delivery. All PI subjects were diagnosed with the vascular pathology based on abnormally high resistance index for GA in the days preceding delivery; the 5 of 12 controls that were tested are indicated.
