# Supplementary material for: Increased Umbilical Cord PAI-1 Levels in Placental Insufficiency Are Associated with Fetal Hypoxia and Angiogenesis
Source: Dis Markers. 2016 Jan 19;2016:7124186. doi: 10.1155/2016/7124186 (PMC4745644; doi:10.1155/2016/7124186)
Supplement: Supplementary file 1 — We sought to determine if the changes in umbilical blood proteins observed were related either to the onset of labor or to the gestational age of the infant. No significant changes were observed for PAI-1, VEGF, or FGF-2 in association with labor in either group. Similarly, umbilical cord oxygen levels were unaffected in our subjects (Supplemental Figure 1). There was also no change in PAI-1 or VEGF associated with GA. There was a significant negative correlation of FGF-2 with GA for controls that could not be seen in IUGR patients (supplemental Figure 2). FGF-2 has previously been reported to gradually decrease in fetal circulation after 20 weeks in normal pregnancy (Hill et al., 1995). Hill, D.J., Tevaarwerk, G.J., Arany, E., Kilkenny, D., Gregory, M., Langford, K.S., and Miell, J. (1995). Fibroblast growth factor-2 (FGF-2) is present in maternal and cord serum, and in the mother is associated with a binding protein immunologically related to the FGF receptor-1. J. Clin. Endocrinol. Metab. 80 :1822–1831. [file 7124186.f1.pdf]

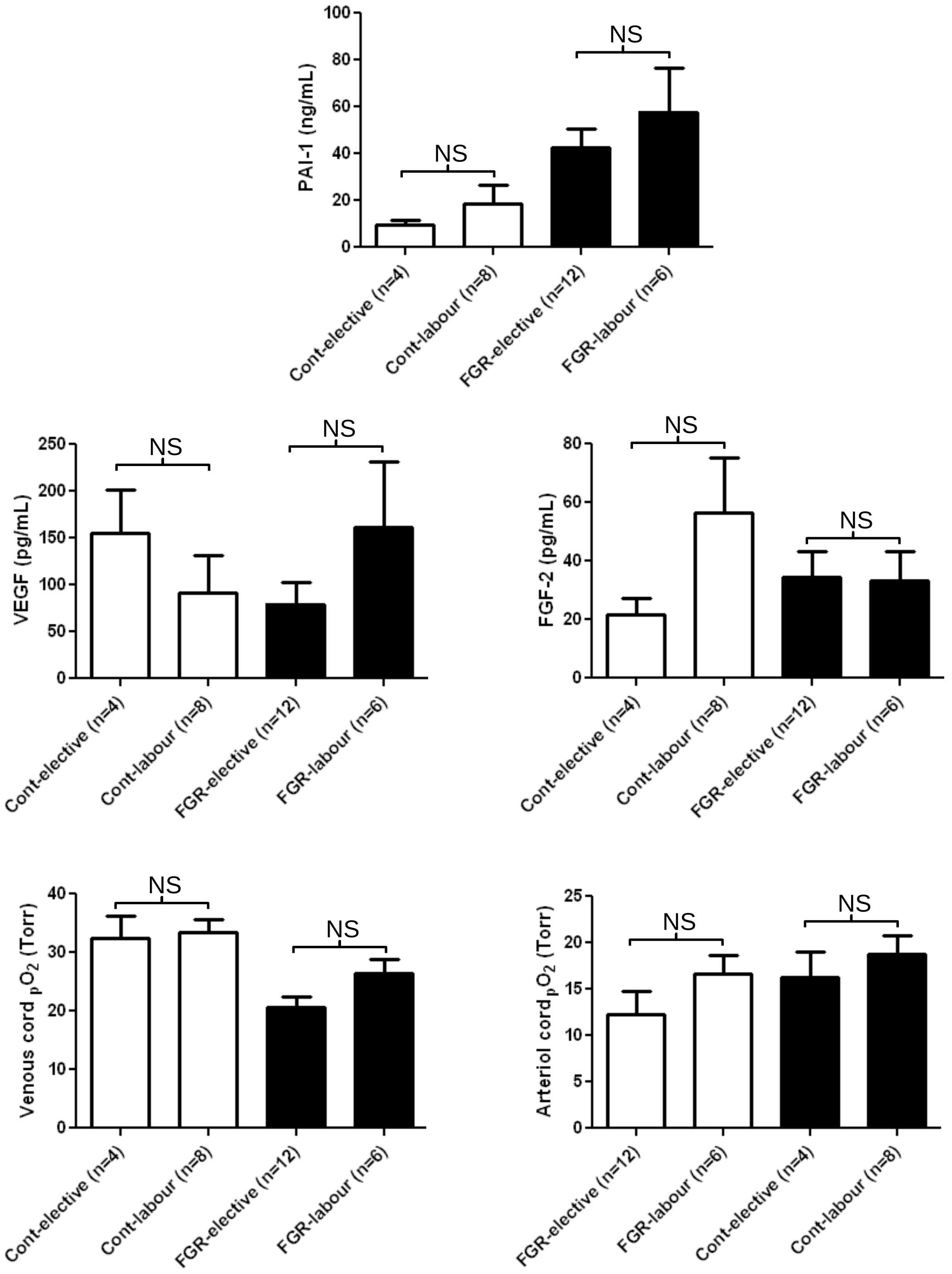

Supplementary Figure 1. **Mode of labor had no effect on protein expression or umbilical blood oxygen.** Levels of umbilical oxygen, PAI-1, total VEGF, and FGF-2 by delivery type (vaginal labor or elective caesarean-section). Separate comparison was made for control and PI samples to determine the effect of delivery type. There was no significant effect of delivery type for either condition. Significance was determined by t-test or Mann-Whitney as appropriate following F-test. Standard error of the mean is indicated.

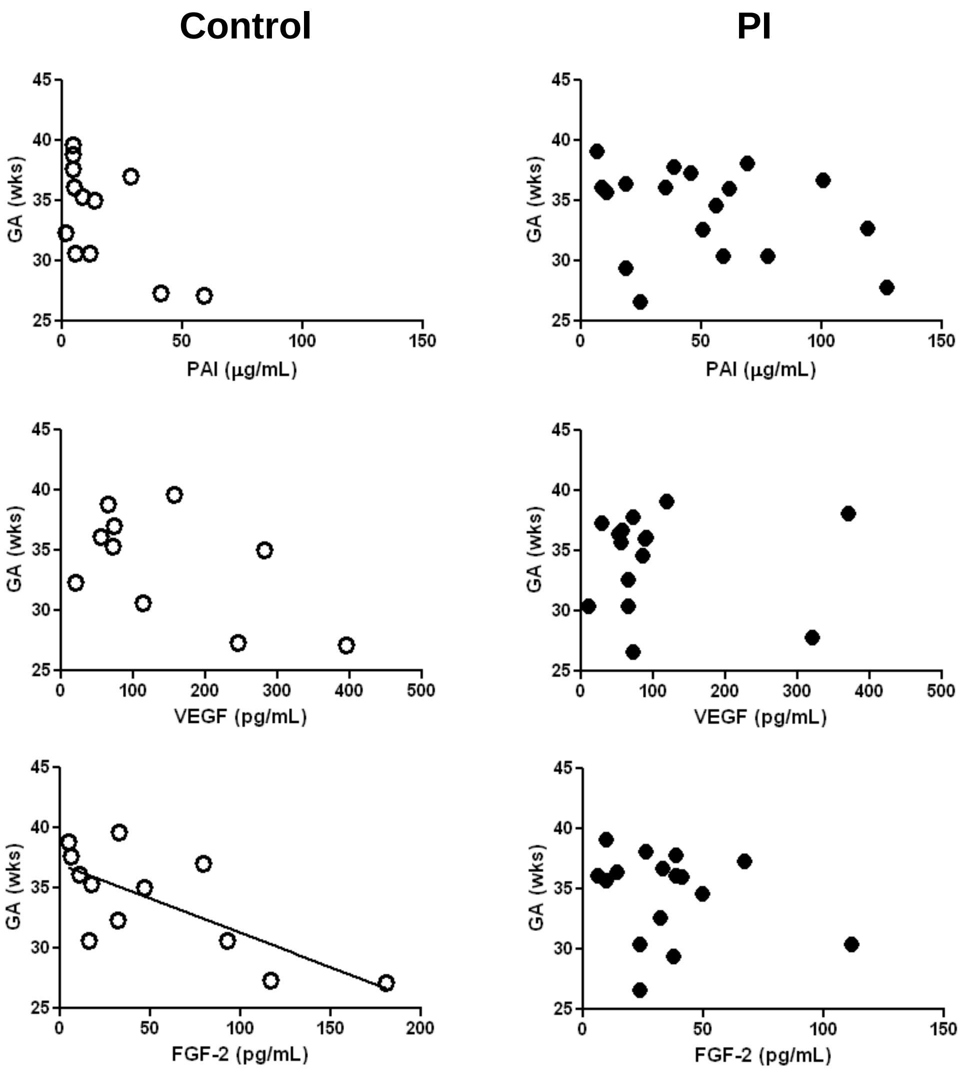

Supplementary Figure 2. **PAI-1 did not change with GA.** Effect of gestational age on PAI-1, VEGF, and FGF-2 levels. Separate Pearson's correlations were made between control (open circles) and PI (closed circle) groups. Only FGF-2 exhibited any relationship, exhibiting a significant negative correlation with gestational age (r=-0.72, p=0.002).
